# Radiation protection necessarily in medical imaging: solution by metal composite 

**Published:** 2019-08

**Authors:** Parinaz Mehnati, Parisa Mehnati, Niloufar Afshin, Reza Malekzadeh

**Affiliations:** ^*a*^Medical Radiation Sciences Research Team, Tabriz University of Medical Sciences, Tabriz, Iran. 2 Department of Medical Physics, School of Medicine, Tabriz University of Medical Science, Tabriz, Iran.; ^*b*^Research Institute for Applied Physics and Astronomy, Tabriz University, Tabriz, Iran.; ^*c*^Student research committee, MD student, School of Medicine, Tabriz University of Medical Sciences, Tabriz, Iran.; ^*d*^Student research committee, Tabriz University of Medical Sciences, Tabriz, East Azerbaijan, Iran.

## Abstract

**Background:**

Using ionizing radiation equipment is known one of the important assays for patient diagnosis. ‎Radiation damage to in field organs was considered as a limitation factor of medical imaging ‎issue but application of the new metal composite shield helped to solving this problem. There ‎are three radiosensitive organs including breast, thyroid and lens that their protection is ‎necessary during chest and skull-neck imaging. In this study radiation dose of sensitive organs ‎during medical imaging and effect of composite shield was presented. ‎

**Methods:**

In this study Bismuth micro- particles as a metal used with Silicon for making composite shield ‎for radioprotection of breast, thyroid and lens. Reduction of radiation dose in the three sensitive ‎organs recorded using Bismuth composite shield. ‎

**Results:**

The findings showed that the dose reduction with bismuth composite shield depends on the ‎scanner, exposure condition and shield design. In skull imaging eyes dose decline varied from ‎‎21% to 50%. In the case of Thyroid, the dose reductions varied from 25% to 84% and finally in ‎chest imaging showed the dose decreasing by 15% to 57% for breast. ‎

**Conclusions:**

Comparison between the eye, the thyroid, and the breast doses in the presence of shield showed ‎that bismuth composite shield has a great potential for sensitive organ protection from radiation ‎damages.‎

**Keywords:**

Ionizing radiation, Medical imaging, Radiation protection, Bismuth composite shield

